# Modulating Dickkopf-1: A Strategy to Monitor or Treat Cancer?

**DOI:** 10.3390/cancers8070062

**Published:** 2016-06-28

**Authors:** Mélody Mazon, Delphine Masi, Madeleine Carreau

**Affiliations:** 1 CHU de Québec Research Center, 2705 Boulevard Laurier, RC-9800, Québec, QC G1V 4G2, Canada; melody.mazon@chudequebec.ulaval.ca (M.M.); delphine.masi@chudequebec.ulaval.ca (D.M.); 2 Department of Pediatrics, Université Laval, Québec, QC G1V 0A6, Canada

**Keywords:** Dickkopf-1, DKK1, Wnt/β-catenin signaling, biomarker, cancer therapeutics, tumour vaccine

## Abstract

Dickkopf-1 (DKK1) is a secreted Wnt/β-catenin pathway antagonist involved in embryogenesis. It was first described 25 years ago for its function in head induction and limb morphogenesis. Since then, this protein has been widely studied in the context of active Wnt/β-catenin signalling during cellular differentiation and development. Dysregulation of DKK1 has been associated with bone pathologies and has now emerged as a potential biomarker of cancer progression and prognosis for several types of malignancies. Reducing the amount of circulating DKK1 may reveal a simple and efficient strategy to limit or reverse cancer growth. This review will provide an overview of the role of Dickkopf-1 in cancer and explore its potential use as a biomarker and therapeutic target.

## 1. Introduction

The Wnt signalling cascade orchestrates a wide variety of biological processes throughout life such as cell division, proliferation, and differentiation [1,2,3]. These cellular processes are required for tissue development and homoeostasis. Wnt signalling is either canonical or non-canonical according to the role played by the effector protein β-catenin. The canonical Wnt/β-catenin pathway is activated following binding of secreted Wnt ligands to their transmembrane receptors of the Frizzled family. This leads to the formation of a cell surface ternary complex composed of a Wnt co-receptor, mainly the low-density lipoprotein receptor-related proteins 5 and 6 (LRP5/6). This protein complex triggers activation of the signalling pathway that, through several cytoplasmic relay components, leads to cytoplasmic accumulation of β-catenin, its translocation to the nucleus and its activation of target genes. To assure a well-organized and timely sequence of events needed for accurate tissue development, Wnt pathways, both canonical and non-canonical are highly regulated. It is therefore not surprising that many types of regulatory proteins have emerged over the course of evolution. These include both intra- and extracellular regulators that modulate activation and repression of Wnts [4]. Of those, the Dickkopf (DKK) family are secreted Wnt/β-catenin antagonists composed of four isoforms, DKK-1 to 4 and one DKK3-related member (DKKL1 or Soggy) [5,6,7]. DKKs, particularly DKK1, antagonize the Wnt/β-catenin signal allowing fine-tuning of signalling events through a feedback loop mechanism. Consequently, dysregulation in any steps of the Wnt/β-catenin signal including DKK1-mediated repression leads to numerous developmental abnormalities and diseases, with the most widely studied being cancer [3,8]. This review will focus on the role of the Wnt/β-catenin pathway antagonist, Dickkopf-1, as a biomarker of cancer and therapeutic target.

## 2. DKK1 Gene Expression and Protein Structure

DKK1’s ability to inhibit the canonical Wnt/β-catenin signalling pathway relies on its capacity to bind simultaneously the transmembrane receptors Kremen-1 or 2 (KRM1/2) and the Wnt/β-catenin co-receptor LRP5/6 [9,10,11,12,13,14]. This extracellular binding leads to endocytosis of the DKK1-associated complex that includes KRM1/2 and LRP5/6 [13,15]. Absence of the co-receptor at the cell membrane impairs a subsequent activation of Wnt/β-catenin signalling. Following internalization, the co-receptor LRP5/6 is recycled to the cell-surface membrane, while DKK1 is shuttled to the lysosome for its degradation. Although the inhibitory functions of DKK1 in the Wnt/β-catenin pathway have been identified first, it was later found that the *DKK1* gene is also a target of Wnt/β-catenin activation [16,17,18]. In fact, the *DKK1* promoter region contains several putative T-cell factor (TCF)-binding sites and was shown to be a direct target of activated β-catenin. In addition, secreted DKK1 could block its own transcription, thus creating a negative feedback loop [17]. Transcriptional repression of *DKK1* has been shown to be mediated by the C-terminal binding protein 1 (CtBP1) in association with the Fanconi anemia C protein (FANCC) [19,20]. Beside TCF/β-catenin, Osterix, an osteoblast-specific transcription factor required for bone formation and differentiation, has been identified as a direct transcriptional activator of the *Dkk1* promoter [21]. Although *DKK1* transcription has been shown to be responsive to other signals and pathways such as apoptotic stimuli or following genotoxic stress, the actual transcription factor directly activating *DKK1* in these cases have not been identified [22,23,24,25,26,27]. Based on public genome databases, the *DKK1* promoter possesses several other putative transcription factor recognition sites and with further study, these may provide new insights on *DKK1*’s regulation.

The human *DKK1* gene maps to chromosome 10q11.2 and encodes a 266 amino acids protein [5,28]. This protein contains six secondary structures: Two alpha helices, four β-sheets, and two highly conserved cysteine-rich domains (CRD-1 and CRD-2) separated by a linker region of variable-length [5,6,29]. The first CRD-1 domain is unique to DKK family members and the carboxy-terminal CRD-2 is highly conserved and folded in a Colipase-like domain containing disulfide bonds and short β-strands [30,31,32]. This C-terminal domain is necessary and sufficient to inhibit canonical Wnt/β-catenin signalling. Various post-translational modifications can be found on DKK1, such as phosphorylation and glycosylation [31]. However, the role of these post-translational modifications in Wnt/β-catenin signalling need to be further explored.

## 3. DKK1 Protein Function in Development

DKK1 was first described as a head inducer in Xenopus during embryogenesis [33]. Subsequently, DKK1 has been detected in other vertebrates, including humans, and in some invertebrates (Dictyostelium, Cnidarians, Urochordates, and Ascidians) excluding Drosophila and Caenorhabditis elegans [34]. Earliest expression of xDkk1 localized in tissues associated with anterior specification such as the Spemann organizer [33]. In mouse embryos, Dkk1 is first detected at 6.5 days post-conception and marks head mesoderm cells [33]. Other developmental models also showed that Dkk1 expression played an important role in head patterning but also showed its involvement in the formation and patterning of different organs and tissues including the eye, heart, limb vertebra, skin and bone [7,29,35]. For instance, *Dkk1* knockdown and knockout models lacked anterior head structures and anomalies in limb formation and digit patterning. To counteract embryonic lethality of the knockout mouse model, a Doubleridge mouse model has been created [36,37]. This viable and fertile model contains a hypomorphic *Dkk1* allele allowing 10% of *Dkk1* expression. This mouse model shows normal head development but postaxial polysyndactyly further supporting the involvement of Dkk1 in digit patterning. Indeed, Dkk1 involvement in limb formation has been strengthened by different Dkk1 overexpression models, in which limb truncation and expansion of the limb growth-controlling zone is observed in chicks. Mechanistically, Dkk1 functions as an inducer of apoptosis of interdigital mesenchymal cells within the cell death zone allowing correct patterning of the digits [24]. Dkk1 seems also implicated in skin development as shown by a hairless phenotype in Dkk1-overexpressing mice, altered melanocyte proliferation and differentiation, decreased melanin transfer from melanocytes to keratinocytes and reduced pigmentation and thickness of the skin [38,39]. Other studies have highlighted a role of DKK1 in bone metabolism. For instance, knockdown and knockout studies have shown an increase bone mineral density whereas Dkk1 overexpression is associated with osteopenia in mice [40,41,42]. In support of these findings, Dkk1 antibody treatments increased trabecular bone volume and bone mineral density. In humans, high DKK1 levels in peripheral blood correlates with a decrease in bone density, while mutations in LRP5 that impede binding to DKK1 result in high bone density [43,44,45].

## 4. DKK1 as a Biomarker of Cancer Initiation and Progression

The first hint that DKK1 could serve as a biomarker of pathophysiologic conditions came from the study of Tian et al. showing abnormally high levels of DKK1 in plasma from patients with multiple myeloma (MM) [45]. In this study, the authors compared expression of a panel of genes in patients with MM in the presence or absence of osteolytic lesions and found elevated levels of DKK1 detectable in peripheral blood of the patients with bone lesions. DKK1 levels in plasma correlated with gene expression pattern of *DKK1* in plasma cells. This study suggested that DKK1 could serve as a marker for MM progression and that cancer mediated modulations of DKK1 influences bone metabolism. This led to an interest in studying DKK1 expression and protein levels in patients with cancers known to induce osteolytic lesions. One such case is osteosarcoma (OS), an aggressive malignant neoplasm, which occurs when mesenchymal cells are transformed. Studies in paediatric patients with OS showed significantly elevated levels of DKK1 in serum of patients with maximal DKK1 expression detected in tumour cells and tumour-surrounding cells [46]. These results support the idea that DKK1 could serve as a diagnostic tool for cancer-mediated bone lesions. In fact, Hall et al. showed that DKK1 plasma levels rise when prostate cancer cells metastasize to bone and induce osteolytic lesions [47,48,49]. Although increased serum DKK1 seemed to be an early event in prostate cancer, which declines as cancer progresses, high DKK1 levels were associated with shorter overall survival suggesting that DKK1 could serve as a cancer prognostic marker [50]. Indeed, this was shown to be the case for patients suffering from triple-negative breast cancer subtypes, non-small cell lung cancer, esophageal squamous cell carcinoma, urothelial carcinoma and bladder cancer, gynaecological cancers or hepatocellular carcinoma (HCC) where high levels of DKK1 correlated with poor survival [51,52,53,54,55,56,57,58,59,60,61]. Other studies also showed elevated levels of DKK1 in patients’ sera or tumour specimen from a squamous cell carcinoma of the head and neck (SCCHN), including esophageal and laryngeal tissues [62,63,64]. These studies combined with those from Peng et al. suggest that measurement of serum DKK1 combined with the presence of autoantibodies against DKK1 could serve as an early screening process for SCCHN, but also as an alternative to invasive screening, notably endoscopic examination followed by histological biopsy [65]. Because the presence of DKK1 in sera correlated with different types of cancers, Sato et al. analysed DKK1 levels in serum samples from 906 patients with cancers of either the pancreas, stomach, liver, bile duct, breast and cervix [66]. The majority of cancer patients presented elevated DKK1 levels compared to healthy controls and thus confirmed previous data supporting the usefulness of DKK1 as a serological biomarker of cancer. In addition, a large-scale multicenter study was performed with patients affected by liver diseases including HCC, chronic hepatitis B virus (HBV) or liver cirrhosis to assess whether DKK1 could serve as an alternative biomarker to alpha-fetoprotein (AFP) for HCC diagnosis [67]. Their results showed that elevated serum DKK1 concentration could help distinguish patients with HCC from those with cirrhosis and HBV infections. However, a recent study showed that AFP remained the best single marker for HCC but in combination with DKK1 had the best diagnostic performance [68]. Together, these studies suggest that levels of DKK1 might serve as a prognostic tool of cancer initiation and progression. However, there are conflicting studies showing down regulation or silenced expression of DKK1 in cancers. These include intestinal and colon cancers where *DKK1* transcriptional silencing is the result of promoter hypermethylation [17,69]. It has also been proposed that *DKK1* silencing in colon cancer suggests a tumour suppressor role for DKK1 and thus the need for its repression to allow tumour growth. This paradoxical behaviour of DKK1 might be explained by its subcellular location and associated function. Indeed, DKK1 nuclear staining was observed in colon cancer cells and associated with increased expression of genes involved in cellular detoxification and survival [70,71]. This nuclear activity of DKK1 correlated with chemoresistance and decreased survival in colorectal cancer patients. Although *DKK1* gene silencing was seen in colorectal tumour cells, Gurluler et al. showed elevated DKK1 levels in serum of patients suffering from stages II and III colon cancer, with higher levels in stage III than in stage II [72]. Increased DKK1 levels in later stages of colon cancer may be indicative of tumour invasion, differentiation and metastasis thus supporting a role of DKK1 as a prognostic factor. Accordingly, a meta-analysis of the prognostic value of DKK1 in gastric cancer showed that DKK1 correlated with tumour invasion and poor survival [73]. These different studies suggest that the detection of elevated DKK1 levels in the plasma of patients correlates with tumorigenesis from various types of tissues. Therefore, DKK1 may be a good candidate protein to monitor cancer both as a diagnostic tool and as a prognostic indicator.

## 5. DKK1 as a Therapeutic Target

Beyond its potential use as a biomarker for several diseases, including cancers, DKK1 is viewed by some as a promising target for cancer therapy. The first evidence showing therapeutic potential of targeting DKK1 has been highlighted by the treatment of bone-related diseases including multiple myeloma, rheumatoid inflammatory disease and disorders or cancers affecting bone metabolism. Several preclinical models of bone diseases including multiple myeloma (MM), osteoporosis and rheumatoid arthritis have shown that *DKK1* knockdown, inhibition or neutralization using specific antibodies improved bone weight, mineral content, mineral density, and volume thus reducing the overall bone erosion [74,75,76,77,78]. In xenograft models of cancers that include multiple myeloma (MM), osteosarcoma, HCC, lung, and prostate cancers, neutralization or inhibition of DKK1 has been successful in inhibiting tumour growth in some cases or tumours burden in others [66,79,80,81]. Specifically, rodent MM models of bone explants loaded with MM cells and treated with humanized anti-DKK1 antibodies (BHQ880) showed reduce myeloma burden and/or bone erosion [76,77,79]. Studies using xenograft models of colon cancer showed opposite results, where reduced tumour growth was observed with overexpression of DKK1 [82]. These different observations suggest a tissue-specific role for DKK1 that could profoundly affect its influence of malignant progression.

Nevertheless, data from these studies clearly demonstrated the beneficial effect of anti-DKK1 treatment for MM and supported its use in clinical trials. Indeed, the BHQ880 anti-DKK1 antibody has been tested in patients with multiple myeloma. To date, results from a Phase Ib dose-escalation study of BHQ880 in combination with anti-myeloma therapy has been published [83]. In this study, 28 patients with relapsed or refractory end-stage MM was recruited, of those, seven completed the proposed 24 cycles of BHQ880. Overall, BHQ880 was well-tolerated and some patients showed clinical benefits. However, results should be interpreted with caution because some patients treated with anti-DKK1 antibodies were also receiving anti-myeloma therapy. Further investigation is warranted in order to establish the efficacy of anti-DKK1 therapy in MM and other malignancies. To this end, other clinical trials with BHQ880 have been completed and tested against MM, breast cancer, renal insufficiency and osteoporosis as listed at Clinicaltrials.gov. Unfortunately, no study results have been posted or published as of this time.

Interestingly, a therapeutic approach based on tumour vaccine therapy showed that stimulating autologous T cells from MM patients with DKK1 peptides as the immunogen was able to specifically target myeloma cells without affecting normal blood cells [84]. Active vaccination against DKK1 in mouse models of MM elicited a specific T cell response against MM cells and efficiently protected mice against myeloma [85]. This pre-clinical study provides a proof-of-concept that DKK1 vaccines could be used as a therapeutic approach against MM as well as for the prevention of recurrent MM. Currently, there are no clinical trials listed or referenced to this approach.

## 6. Conclusions

The DKK1 protein has lately become a focus of attention in cancer research, both as a biomarker and potential therapeutic target. Mechanisms leading to upregulation of DKK1 in many cancers are beginning to emerge. For instance, some have highlighted a role of DKK1 in cancer growth through upregulation of stress response genes, such as the *Aldehyde dehydrogenase 1A1*, which is involved in detoxification of chemotherapeutic agents. This mechanism would predict chemoresistance in patients where levels of DKK1 are found elevated. The fact that many different cancers provoke an overexpression of this protein in the blood of patients makes it a promising tool for the clinic.
